# Should COVID-19 be branded to viral thrombotic fever?

**DOI:** 10.1590/0074-02760200552

**Published:** 2021-04-30

**Authors:** Rubens Carmo Costa-Filho, Hugo Caire Castro-Faria, José Mengel, Marcelo Pelajo-Machado, Marco Aurélio Martins, Érica Távora Leite, Hugo Tannus Mendonça-Filho, Tatiana de Arruda Campos Brasil de Souza, Gonzalo Bentacor Bello, José Paulo Gagliardi Leite

**Affiliations:** 1 Hospital Pró-Cardíaco, Américas Serviços Médicos, United-Health Group, Rio de Janeiro, RJ, Brasil; 2 Fundação Oswaldo Cruz-Fiocruz, Instituto Oswaldo Cruz, Laboratório de Imunofarmacologia, Rio de Janeiro, RJ, Brasil; 3 Fundação Oswaldo Cruz-Fiocruz, Instituto Oswaldo Cruz, Laboratório de Imunologia Clínica, Rio de Janeiro, RJ, Brasil; 4 Centro Universitário Arthur Sá Earp Neto, Faculdade de Medicina de Petrópolis, Petrópolis, RJ, Brasil; 5 Fundação Oswaldo Cruz-Fiocruz, Instituto Oswaldo Cruz, Laboratório de Patologia, Rio de Janeiro, RJ, Brasil; 6 Fundação Oswaldo Cruz-Fiocruz, Instituto Oswaldo Cruz, Laboratório de Inflamação, Rio de Janeiro, RJ, Brasil; 7 Instituto Nacional de Câncer, Rio de Janeiro, RJ, Brasil; 8 Políticas de Saúde do United-Health Group, Rio de Janeiro, RJ, Brasil; 9 Fundação Oswaldo Cruz-Fiocruz, Instituto Carlos Chagas, Laboratório de Proteômica Estrutural e Computacional, Curitiba, PR, Brasil; 10 Fundação Oswaldo Cruz-Fiocruz, Instituto Oswaldo Cruz, Laboratório de AIDS e Imunologia Molecular, Rio de Janeiro, RJ, Brasil; 11 Fundação Oswaldo Cruz-Fiocruz, Instituto Oswaldo Cruz, Laboratório de Virologia Comparada e Ambiental, Rio de Janeiro, RJ, Brasil

**Keywords:** endothelium, COVID-19, SARS-CoV-2, SARS CoV Mpro, inflammation, coagulation, hypercoagulability, thrombosis, thromboelastometry

## Abstract

Coronaviruses can cause a diverse array of clinical manifestations, from fever with symptoms of the common cold to highly lethal severe acute respiratory syndrome (SARS) and middle east respiratory syndrome (MERS). SARS-CoV-2, the coronavirus discovered in Hubei province, China, at the end of 2019, became known worldwide for causing coronavirus disease 2019 (COVID-19). Over one year’s time period, the scientific community has produced a large bulk of knowledge about this disease and countless reports about its immune-pathological aspects. This knowledge, including data obtained in postmortem studies, points unequivocally to a hypercoagulability state. However, the name COVID-19 tells us very little about the true meaning of the disease. Our proposal is more comprehensive; it intends to frame COVID-19 in more clinical terminology, making an analogy to viral haemorrhagic fever (VHF). Thus, we found irrefutable evidence in the current literature that COVID-19 is the first viral disease that can be branded as a viral thrombotic fever. This manuscript points out that SARS-CoV-2 goes far beyond pneumonia or SARS. COVID-19 infections promote remarkable interactions among the endothelium, coagulation, and immune response, building up a background capable of promoting a “thrombotic storm,” much more than a “cytokine storm.” The importance of a viral protease called main protease (Mpro) is highlighted as a critical component for its replication in the host cell. A deeper analysis of this protease and its importance on the coagulation system is also discussed for the first time, mainly because of its similarity with the thrombin and factor Xa molecules, as recently pointed out by structural comparison crystallographic structures.

About the SARS-CoV-2

SARS-CoV-2 is a novel coronavirus, the seventh one in alfa and beta-coronaviruses (CoVs), responsible for the coronavirus disease 2019 (COVID-19) pandemic as declared by the World Health Organization (WHO) on March 11, 2020.^1^ COVID-19 is by far the most significant global calamity since the Second World War, probably better defined as a syndemic due to the combination of severe health problems and socioeconomic drawback, which has strongly accentuated inequalities worldwide.^2^ ^,^ ^3^ On February 23th, COVID-19 statistics reported by the Centre for Systems Science and Engineering (CSSE) at Johns Hopkins University (JHU) displayed 111.974.905 worldwide cases of the disease and over 2.481.140 deaths involving 192 countries, Brazil being the second one most affected country in the global ranking with 247.143 deaths.^4^ On February 21th, COVID-19 statistics reported by the CSSE at JHU displayed 111.251.603 worldwide cases of the disease and over 2.463.735 deaths involving 192 countries, Brazil being the second one most affected country in the global ranking with 245.977 deaths

Researching targets

Several viral proteins have been prioritised as SARS-CoV-2 antiviral drug targets, such as the spike protein, the RNA-dependent RNA polymerase (RdRp), the main protease (Mpro), and the papain-like protease (PLpro).^5^ Indeed, the SARS-CoV-2 genome encodes four structural proteins, sixteen non-structural proteins (NSPs) that carry out crucial intracellular functions, and nine accessory proteins.^6^ In addition to the proteins mentioned previously above, the virus needs a foreign protein, the TMPRSS2 (Transmembrane Protease Serine 2), to infect the host’s cells. Thus, the TMPRSS2 protein is considered an important therapeutic target, creating the potential for infection prevention if inhibited at the early stages of the infection. SARS-CoV and other coronaviruses also use TMPRSS2 for their S protein activation, and the protease is expressed in SARS-CoV target cells throughout the human respiratory tract and other organs.^7^ ^,^ ^8^ ^,^ ^9^

Is the Mpro a crucial molecule to battle against SARS-CoV-2 infection?

The viral protease Mpro is one of the most suitable targets for therapeutic strategies against SARS-CoV-2. The intracellular translation of the viral RNA results in the synthesis of two critical polyproteins, which are processed by two virally encoded cysteine proteases, a papain-like protease - PLpro and a 3CL-like protease - 3CLpro (also alluded to as NSP5 or as Mpro), from now on referred as SARS-CoV-2 Mpro protease. In the investigational article written by Sinthyia Ahmed et al.,^5^ it was verified significant binding affinity and interaction of 76 prescription drugs against RNA dependent RNA polymerase (RdRp) and Mpro of SARS-CoV-2. Mengist et al.^10^ described that inhibitors of the Mpro prevent the replication of SARS-CoV-2 *in vitro*. This effect is explained by the role of Mpro cleaving polyproteins (pp1a and pp1ab) translated from virus genomic RNA, yielding non-structural proteins that are necessary for assembling the viral replication transcription complex (RTC) for viral RNA synthesis in the host’s cells. Any inhibitor that could cross the cellular membrane, in a non-toxic concentration to the host, and is capable of binding to Mpro inhibiting its activity as a virus protease will undoubtedly impair virion assembly and the release of the new intact virion. In summary, coronaviruses Mpro molecule is fundamental to facilitate viral assembly by cleaving polyproteins and is a suitable target to stop viral reproduction. Additionally, shortly, studies using this protease’s crystal structures will provide a foundation for designing potent inhibitors of Mpro for clinical use, giving this dreadful disease an expeditious end.^7^ ^,^ ^10^ ^,^ ^11^ ^,^ ^12^ ^,^ ^13^ ^,^ ^14^ A consortium of scientists is trying to speed up developing a viral protease inhibitor with a massive crowdsourced initiative to combine the expertise of multiple labs and researchers worldwide and process as many possible protease inhibitor structures as possible. Earlier in March, crystallographers at the Diamond Light Source also solved the SARS-CoV-2 main protease structure at high resolution.^15^ ^,^ ^16^ Scientists hope to find several lead compounds by using artificial intelligence (AI) algorithms to suggest changes to the molecules, especially SARS-CoV-2 Mpro, to help speed up drug development.^17^

Does endothelial dysfunction have an essential role in SARS-CoV-2 infection?

The endothelium functions as a receptor-effector organ, responding to the different physical or chemical stimuli by secreting specific mediators. It may maintain the vasomotor balance and vascular tissue homeostasis. Additionally, there are different types of endothelium in the human body - some expressed more tissue-type plasminogen activator (t-PA), such as those blood vessels in the heart, others more thrombomodulin (TM), such as the blood vessels in the lungs.^18^ ^,^ ^19^ ^,^ ^20^ The endothelium is no longer perceived as a layer of inert coating within the vessels, but more as the conductor who orchestrates multiple functions, playing a central and critical role in many physiological processes, including:^21^

- Vasomotor tone;

- Transport of blood cells and underlying tissues;

- Maintenance of blood flow;

- Permeability;

- Angiogenesis;

- Innate and adaptive immunity.

Thus, the endothelium is an active paracrine, endocrine, and autocrine organ, and it is fundamental for the regulation, maintenance, and equipoise in vascular health.^22^ The endothelium is made up of layers of cells that line the vessels. By itself, it weighs approximately 1 kg in an adult of average size and weight (70 kg), and it could cover an area of 4,000 to 7,000 m^2^.^19^ If stretched out, these cells would make four turns around the planet, extending its length for about 180,000 km, giving an insight into the magnitude of the problem when that system goes into dysfunction. Moreover, the endothelium presents different structures and functions, as discovered in 1966, through Florey’s ultrastructural observations.^23^ It has an astonishing functional heterogeneity highlighted when specific subsets of blood vessels are analysed.^24^ It is important to emphasise that the endothelium is also modulated in different diseases and infections. Initial symptoms of viral haemorrhagic fever (VHF) are remarkably similar to the SARS-CoV-2 condition. For instance, acute VHF infections start with fever, myalgia, and malaise with progressive prostration, typically lasting for three to four days when vascular manifestations begin to emerge, including vascular permeability and small-vessel damage. The vascular endothelium infection may be familiar to all VHFs,^25^ and the consequent endothelial dysfunction is fundamental in the genesis of bleeding, which is not universal but is often seen when thrombocytopenia, or severe platelet dysfunction, are detectable. Otherwise, in SARS-CoV-2, clinical reports increasingly suggest a significant injury on the endothelial cells, but with the uncommon presence of thrombocytopenia.^25^ ^,^ ^26^ The bleeding in VHF seems to be diffuse and due to capillary damage. On the other hand, in COVID-19, endothelial infection and consequent endotheliitis have startled the medical community presenting itself as an intense hypercoagulability disorder causing diffuse micro-thrombosis,^27^ ^,^ ^28^ that in fact, it may be associated with robust thrombin generation.

Coagulation disturbances in coronaviruses infections

Recently, an observational study was conducted by Klok et al.,^29^ involving 184 COVID-19 in intensive care unit (ICU) patients in three Dutch hospitals to investigate the incidence of thrombotic complications. This study verified 31 cases of thrombosis in the setting of critical care, with 81% developing a pulmonary embolism, 3.2% deep venous thrombosis, 6.4% were identified with catheter-related upper extremity thrombosis, and 9.6% with ischemic stroke.

These findings made the authors consider that rather than treating all COVID-19 patients in the ICU with therapeutic anticoagulation, physicians should be vigilant for signs of thrombotic complications and order appropriate diagnostic tests at a low threshold. Based on very recent brief reports,^30^ ^,^ ^31^ ^,^ ^32^ ^,^ ^33^ retrospective observational studies,^34^ ^,^ ^35^ and one prospective cohort study, it has been shown that 64 out of 150 patients developed thrombosis in the ICU, and 16% of those that suffered from thrombosis, had a pulmonary embolism.^36^ Another controversial retrospective study^37^ that stratified patients based on sepsis-induced coagulopathy (SIC) score, and D-dimer levels, suggested those who meet the International Society of Thrombosis and Haemostasis (ISTH). Sepsis-induced coagulopathy, or existing markedly elevated D-dimer, may benefit from anticoagulation, especially low molecular weight heparin (LMWH). Based on this, the authors suggested that the use of heparin would decrease mortality in severe SARS-CoV-2 infections, showing no evidence of thromboses such as deep venous thrombosis or pulmonary embolisation.

At the end of March 2020 - The ISTH launched recommendations and guidelines to recognise and handle this dreadful and profound hypercoagulable state caused by COVID-19 immunothrombosis.^38^ Based on the available literature, the ISTH counseled to measure the D-dimers, prothrombin time, and platelet count in all patients who present with COVID-19 illness. However, case descriptions have emerged in the literature where the presence of antiphospholipid syndrome (APL)^30^ and lupus anticoagulants (LA) has been found, which may otherwise affect laboratory aPTT prolonging it. Thus, this could hinder clinical judgment for the use of prophylactic or therapeutic heparin doses.^39^ Interestingly, the authors verified that most (91%) in-patients with COVID-19 with a prolonged aPTT were positive for lupus anticoagulant. The authors also identified that these patients had an associated factor XII deficiency, which implies a thrombosis tendency. It’s worth noting that the adjective “catastrophic” was added to the term antiphospholipid syndrome (APS) in 1992 by Ronald Asherson, highlighting a sped-up form of this syndrome resulting in multiorgan failure.^40^ Furthermore, approximately 60% of the catastrophic episodes are preceded by a precipitating event, mainly infections.^41^ Systematic reviews and meta-analyses have evaluated the role of infections, particularly viral infections, on aPL and thromboembolic circumstances. The first included studies in patients with HIV-1, HCV, and HBV, and the second reported on patients with HCV or HBV.^42^ To our knowledge, catastrophic antiphospholipid syndrome (CAPS), also Known as Asherson’s syndrome related to COVID-19, was not described until the present moment, and further investigation will be necessary.

The interplay between thrombin and the endothelium

Hemostasis is a defense mechanism that protects the organism from the loss of blood by plugging injured vessels.^43^ ^,^ ^44^ If this protective mechanism goes amiss, abnormal haemostasis may lead to bleeding disorders or, if it occurs in excess inside the blood vessel or in the wrong location, it may cause life-threatening thrombosis, partially or entirely blocking the vessel.

Hemker^45^ formulated by 14 years ago a very shrewd assertion “The more thrombin, the more thrombosis but, the less bleeding, the less thrombin, the less thrombosis but, the more bleeding.”. To address *Hemker’s Law*, it is necessary to consider how the activity of thrombin is controlled by its structure. Once bound as the thrombin-thrombomodulin complex, it redirects its substrate specificity from procoagulant to anticoagulant reactions or even to antifibrinolytic action by blocking the plasmin action by TAFI reaction (Fig. 1). This “anticoagulant profile” could be accelerated by substrates, such as platelet factor 4 (PF4) secreted out of platelet granules during its activation or the glycosaminoglycans heparin and heparan sulfate available over the normal endothelium in the glycocalyx. Moreover, endothelial cell phenotypes vary in space and time.^19^ According to this information, monitoring only thrombin generation would not mirror what is happening over the endothelium, which may react in different ways and magnitudes.^46^

**Fig. 1:**
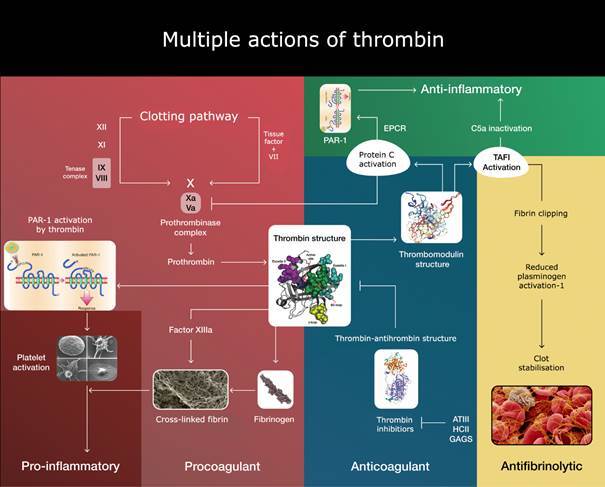
thrombin is a multifaceted serine protease that exerts multiple functions depending on its ligands. Also, the complexity of thrombin’s regulatory systems lies in the dynamics of its two exosites. The exosite I not only link fibrinogen to cleave it but even start the fibrin formation. When thrombin is bound to thrombomodulin, this complex can activate protein C thousands of times, which stops its generation by negative feedback. Further control occurs at exosite II, where both heparin and antithrombin III act to inhibit thrombin forming complexes.^46^ Moreover, the allosteric effects of thrombin are managed by a sodium ion, which increases thrombin’s activity (not shown in the figure). Thrombin is a potent activator of the platelets. Through PAR-1 and PAR-2 and in a “thrombin storm,” endothelial cells could be more injurious rather than a “cytokine storm” recently remarked to happen in severe acute respiratory syndrome-coronavirus 2 (SARS-CoV-2).^75^ Excess of thrombin generation and endotheliitis with the extraordinary formation of neutrophil extracellular traps release (NETosis) could carry out a colossal thrombosis amplification.^76^

Another intriguing similarity is that growing evidence suggests that SARS-CoV-2, like Ebola or Dengue viruses, seems to trigger neutrophil-induced immunopathogenesis. COVID-19 severity is also associated with neutrophil over-expression of neutrophil extracellular traps (NETs).^47^ ^,^ ^48^ NETs release (NETosis) (Fig. 2) is a powerful mechanism of microbial destruction by which neutrophils die and release digestive granules containing neutrophil elastase (NE) and myeloperoxidase (MPO),^49^ ^,^ ^50^ which amplifies immunothrombosis and inflammation.^51^

**Fig. 2:**
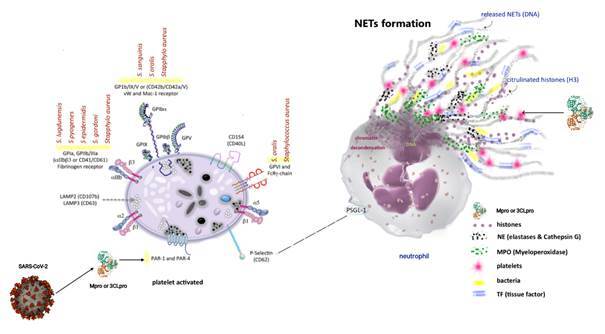
a schematic representation is modified according to reference^51^ of how the interplay between neutrophils and platelets is important to neutrophil extracellular traps release (NETosis). Also, examples of interaction over the platelet’s receptors by a vast array of viruses and bacteria. Herein, a hypothetical action by viral serine protease main protease (Mpro) precisely onto PAR-1 and PAR-4 due to its similarity with thrombin molecules.

Rotational thromboelastometry (roTEM®) could be of value to identify patients with COVID-19 in a hypercoagulability state?

Rotational thromboelastometry (roTEM), a methodology based on thromboelastography (TEG) described initially by Hartert in 1948, is used at the bedside to rapidly access (ex-vivo) viscoelastic properties of the blood in various kinds of situations. roTEM^®^ is designed to verify the interactions of platelets, cells, and the coagulation factors from initial platelet-fibrin interaction, through platelet aggregation, clot strengthening, and fibrin cross-linking to fibrinolysis if it happens. The roTEM system is already utilised in several clinical settings, e.g., for monitoring of hemostasis during liver transplantation, cardiac surgery, trauma, extra-corporeal membrane oxygenation (ECMO), burns, coagulopathy, and intensive care, and has been shown to be a point of care device for rapid diagnosis and differentiation of hyper- and hypocoagulability and hyperfibrinolytic scenario.^52^ ^,^ ^53^ ^,^ ^54^ ^,^ ^55^ ^,^ ^56^ Recently, many reports investigating the use of thromboelastometry in SARS-CoV-2 were published. All of them described the presence of hypercoagulability patterns, especially in patients with increased disease severity and inflammation.^57^ ^,^ ^58^ ^,^ ^59^ Severe elevation of D-dimer seems to be a hallmark of mortality in COVID-19.^60^ Raza et al.^61^ showed that only 5% of trauma patients developed severe fibrinolysis on roTEM analysis with a median D-dimer level of 39,687 ng/dL and that only 1.84% of fibrinogen (mean 210 mg/dL) was converted to D-dimer. In contrast, Madathil et al.^57^ revealed that the D-dimer level in COVID-19 patients was 15,465 ng/mL, but only 0.21% was converted from fibrinogen (mean 734 mg/dL), altogether with roTEM profile of hypercoagulability. Taken together, those patients with severe COVID-19 demonstrated significant elevation of D-dimer not because of fibrinolysis as well described in trauma patients, but instead by the widespread of microthromboses and endotheliitis. Importantly, Pavoni et al.^58^ described a hypercoagulability state that persisted over time in a retrospective study of critically ill COVID-19 patients admitted to ICU.

The vast amounts of publications describing the undisputed presence of thrombosis recently observed in postmortem studies of severe cases of COVID-19 and even in patients who already had received hospital discharge reinforce our argument.^62^ ^,^ ^63^ ^,^ ^64^ ^,^ ^65^ With all that in mind, we can certainly infer that thromboelastometry could help physicians at the bedside to speed up monitoring, to take action, and to follow up on the evolution of this deadly disease.

Coagulation modifiers targeting SARS-CoV-2 main protease

Recently, a notable study by Biembengut and Souza Brasil^11^ has investigated potential inhibitors of the Mpro using crystallography structures of the proteases, such as published by Zhang et al.^66^ Afterward, one solution of the Mpro tridimensional structure allowed investigators to find potential viral replication inhibitors. These authors bring to our knowledge (after screening 4,334 compounds from the Drugbank^®^ Database)^67^ some new remarkable discoveries. These authors stressed out, as many others did,^12^ ^,^ ^14^ ^,^ ^66^ the potential for inhibitors of SARS-CoV-2 Mpro as antiviral drugs (Fig. 3). It’s worth mentioning that a vital observation drawn from their results is that some of these molecules were anticoagulant drugs. Furthermore, Brazilian authors recently reported that superposing Mpro with factor Xa and thrombin structures depicted minor values of root-mean-square deviation (RMSD), the average distance between the atoms; in other words, these molecules have too high structural similarity among them (Fig. 3). In that study, the Mpro RMSD values with factor Xa and thrombin were 2.75Å and 2.49Å, respectively, showing fold conservation. Both thrombin and Mpro are proteases. Thrombin cleaves mainly Agr-Gly sites, and Mpro shows sequence promiscuity.^68^ Based on this information, it is coherent to surmise that Mpro that exists inside the host’s cell when the viruses are replicating could be released upon cell death in the blood circulation and act as an additional source procoagulant capable of inducing or synergising with endogenous factors in thrombogenesis. All of these could give grounds for augmenting endotheliitis. Perhaps more generation of prothrombinase/tenase complexes, secondary to a downregulation of the anti-inflammatory effects of the thrombomodulin/protein C/EPCR (endothelial protein C receptor) system, yields unhealthier endothelium and NETosis leading to a massive state of hypercoagulability and inflammation. By saying that, some questions emerge:

**Fig. 3:**
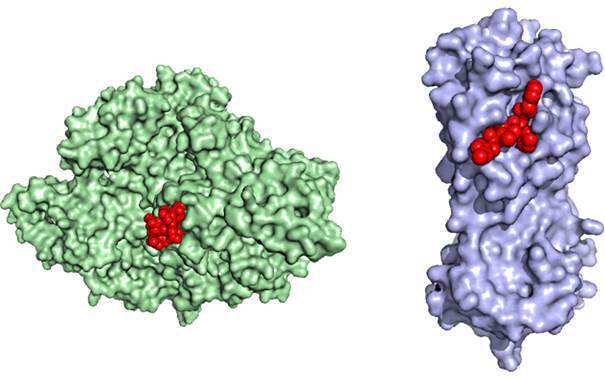
the crystal structures of RNA-dependent RNA polymerase (RdRp) (green) and main protease (Mpro) (magenta) surface like the paper published by Sinthyia Ahmed et al.,^5^ employing a repurposing approach to identify drugs as candidates for binding without altering their native protein structure, describing the docking location (in red) of molecules with the same affinity to Ser, Ala, Gly (Serine protease). This Crystal structure graphic was kindly provided by Tatiana Brasil de Souza.^11^

- Does the Mpro viral protein work better or worse than thrombin over all the coagulation factors?

- Does the Mpro act like thrombin over the protease-activated receptors 1 and 4 (PAR-1 and PAR-4) in platelets, justifying the platelet activation in this syndrome? (Fig. 2)

- Could Mpro be tangled with the NET formation, causing more injury to the cells? (Fig. 2)

- Could Mpro be complexed with antithrombin (like thrombin forming thrombin/antithrombin complexes -TATc), causing its sequestration by removing this vital natural anticoagulant already compromised in endotheliitis?

- Could Mpro amplify the accessory pathway of coagulation, complement system, and cause further thrombin activation?

- Thrombin is a natural inhibitor of the ADAMST-13 (**a d**isintegrin **a**nd **m**etalloproteinase with a **t**hrombospondin type 1 motif, member **13**), also known as von Willebrand factor-cleaving protease (VWFCP), very important to the health of the microcirculation. Could Mpro likewise inhibit ADAMST-13 and contribute to increased VWF levels and, hence amplifying micro-thrombosis?

These suppositions raised from vast amounts of publications describing an immense presence of thrombosis recently observed in post mortem and during the severe cases SARS-CoV-2 in critical care and even those that already had received hospital discharge.^63^ ^,^ ^65^ ^,^ ^69^ ^,^ ^70^ In this context, Biembengut and Souza Brasil^11^ identified important coagulation modifiers among 80 compounds with Mpro inhibitor potential. The first was Argatroban; a direct thrombin inhibitor successfully used to treat HIT. The second was three NOACs (Novel Oral Anticoagulants) - Edoxaban, Betrixaban and Apixaban; all are direct factor Xa inhibitors. Apixaban presented a higher theoretical affinity for SARS-CoV-2 Mpro (7.0 kcal/mol) than Factor Xa (5.1 kcal/mol). This finding highlights the potential of NOACs as a weapon against widespread hypercoagulability syndrome but also as antiviral therapies preventing viral replication by inhibiting Mpro within the host’s cell or when it’s released from infected, damaged cells.

Limitations

It is important to say that our purpose in this opinion was to describe possible phenomenological mechanisms that can help in understanding the reasons why thrombotic phenomena are such a central hallmark of COVID-19. It’s not our intention to generate or recommend therapeutic actions. Three international partners have come together in an unprecedented collaboration resulting in multiple platforms randomised controlled trials, REMAP-CAP, ACTIV-4, and ATTACC (NTC 02735707, 04505774, and 04372589, respectively) to answer important questions related to antithrombotic administration in the COVID-19 setting. A recent clinical guideline from the American Society of Haematology has been published on the use of anticoagulation for thromboprophylaxis in patients with COVID-19.^71^ We still have very low certainty in the evidence to use prophylactic-intensity over intermediate-intensity or therapeutic-intensity anticoagulation for patients with COVID-related critical illness or even acute illness which does not have a suspicion or confirmed VTE. Another example could be drawn from heparin-induced thrombocytopenia (HIT) patients, where HIT-antibodies formed (IgG/PF4/heparin) can activate endothelium, macrophages, and platelets surfaces (CD32) through FcYRIIa receptors, exacerbating inflammation. The presence of HIT in severe COVID-19 is a complex issue^72^ ^,^ ^73^ ^,^ ^74^ beyond the scope of this opinion. Association between COVID-19, autoimmunity, and vasculitis remains unknown. Questions are raised if the antiphospholipid antibodies could, in some way, exacerbate thrombosis or even amplify endotheliitis already in the course of SARS-CoV-2 infection.

In Conclusion

Based on the findings discussed herein, it is reasonable to propose that COVID-19 should be branded as the first VTF. That would be more informative not only from a clinical standpoint but also from a public perception point of view. Nomenclatures such as SARS-CoV-2 or COVID-19 hide an enormous spectrum of this disease since it has been coined in times of lesser knowledge. Today, it can be said that we have a wider view of this multifaceted syndrome, its progression, and sequelae, much beyond lung injuries. Therefore, we leave the suggestion to brand COVID-19 as VTF, which in fact, it has already been proved to be.
